# Integrative computational approaches, molecular docking, and dynamic simulations reveal the antimycobacterial activity of fisetin as a potential inhibitor of *Mycobacterium tuberculosis*

**DOI:** 10.1007/s10822-026-00786-6

**Published:** 2026-04-10

**Authors:** Sajjad Ahmed Khan, Muzafar Ahmad Rather, Ziyi Jia, Muhammad Umer Khan, Syed Mehmood Qadir, Niaz Ahmed, Hasan Ejaz, Muharib Alruwaili, Anthony D. Baughn, W. Thomas Shier, Muhammad Sheeraz Ahmad

**Affiliations:** 1 University Institute of Biochemistry and Biotechnology, PMAS-Arid Agriculture University Rawalpindi, Rawalpindi, 46300 Pakistan; 2 Department of Medicinal Chemistry, College of Pharmacy, University of Minnesota, Minneapolis, MN 55455 USA; 3 Department of Microbiology & Immunology, University of Minnesota Medical School, Minneapolis, MN 55455 USA; 4 Institute of Molecular Biology and Biotechnology, The University of Lahore, Lahore, 54000 Pakistan; 5 Department of Clinical Laboratory Sciences, College of Applied Medical Sciences, Jouf University, Sakaka, 72388 Saudi Arabia; 6 National Reference Laboratory for Tuberculosis, National TB Control Program, Islamabad, 44000 Pakistan

**Keywords:** Mycobacterium tuberculosis, Fisetin, Molecular docking, MD simulations

## Abstract

The development of effective antitubercular drugs is necessary because tuberculosis remains a major global healthcare burden. To examine the antimycobacterial properties of fisetin, the current study used an integrated method. The in vitro test against *Mycobacterium tuberculosis* H37Ra demonstrated notable inhibitory effects, with an MIC of 100 µg/mL and an MBC of 200 µg/mL. In silico ADMET analysis was used to assess the drug-like characteristics and advantageous pharmacokinetic parameters to explore its molecular mechanism. Significant binding affinities were observed in docking studies against eight core proteins of *Mtb*, with protein kinase B (PknB), a crucial *Mtb* regulator of cell division and survival, showing the highest affinity. Fisetin’s electron stability and reactivity were revealed by DFT research, indicating that it would be able to bind to biological targets. Specifically, the stability of the fisetin-PknB complex was verified by 100-ns-scale molecular dynamics analysis, which maintained its structure and dynamics. Fisetin was shown to have similar or even better binding qualities than the anti-tubercular medication isoniazid when those were compared using docking and MD analysis. Overall, fisetin is a potential multitarget antitubercular drug that merits additional experimental validation using an integrative approach.

**Graphical abstract:**

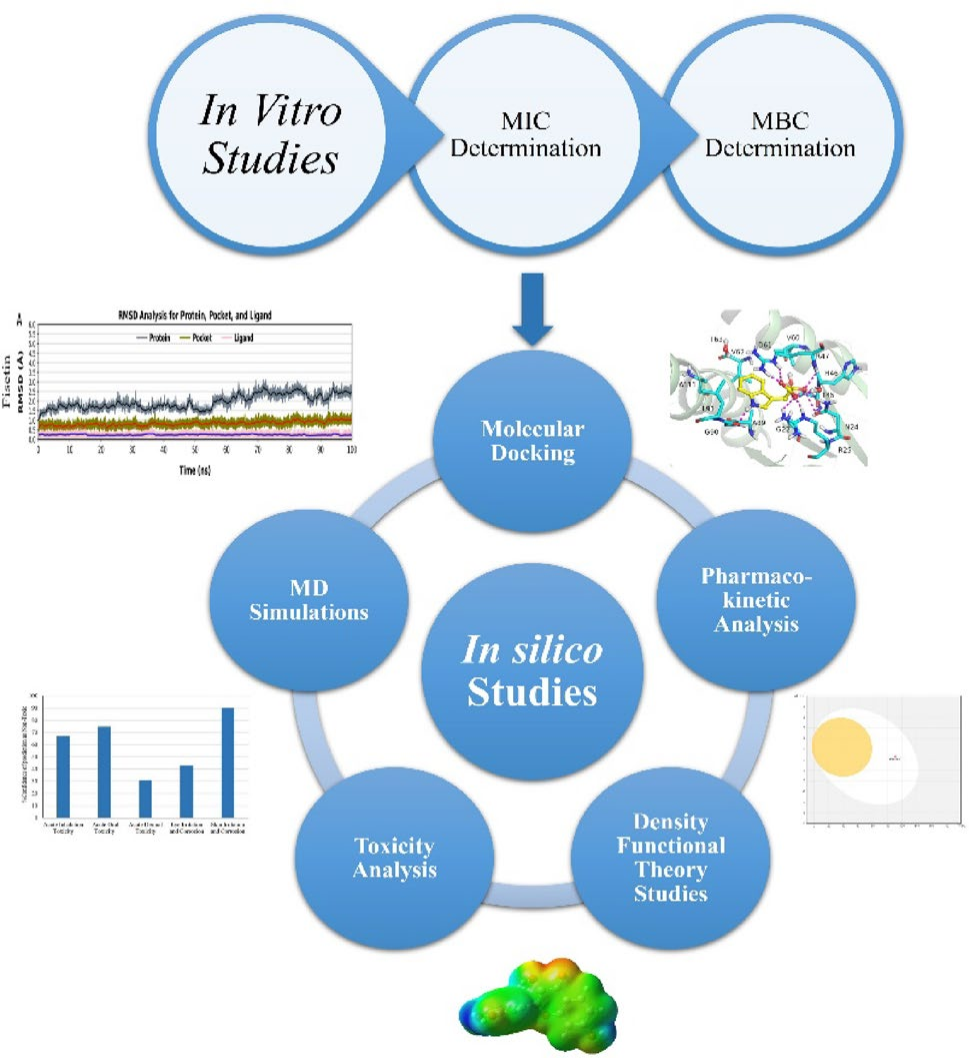

**Supplementary Information:**

The online version contains supplementary material available at 10.1007/s10822-026-00786-6.

## Introduction

*Mycobacterium tuberculosis* (*Mtb*), a rod-shaped member of the Mycobacteriaceae family, is the primary cause of death globally and causes tuberculosis (TB) in humans. TB is the most deadly infectious disease, with approximately 10 million new cases reported in 2017. A lifetime risk of developing active TB exists for an estimated 1.7 billion people with latent TB [1]. TB rates are particularly high in developing countries, where it significantly strains healthcare systems, along with HIV/AIDS and malaria. The prolonged course of several medicines used in traditional TB treatment frequently results in negative side effects and low patient adherence [2]. Drug-resistant tuberculosis can be prevented with a variety of antibiotic regimens; however, long-term use of these drugs raises the risk of drug-resistant strains due to their toxicity [3]. As a result, extensively drug-resistant (XDR) and multidrug-resistant (MDR) *Mtb* strains have emerged. Extrinsic and intrinsic variables are the two main causes of *Mtb* drug resistance. While intrinsic variables are associated with genetic changes in genes responsible for drug targets or the enzymes involved in drug activation, extrinsic factors are related to the total TB population and the efficacy of TB preventive and control strategies within a community [4, 5]. The increasing prevalence of resistant strains is a major obstacle in the treatment of resistant strains [6, 7]. Consequently, there is an urgent need for new, inexpensive antituberculosis medications that work in diverse ways and are less likely to cause resistance.

Animals, microorganisms, marine species, and medicinal plants have shown promise as sources of bioactive chemicals with therapeutic potential. Drug development has greatly benefited from natural products, especially secondary metabolites, which offer a wealth of new lead molecules or pharmacophores for medicinal chemistry intended to cure human ailments [8]. Natural products and their derivatives have shown potential for treating numerous infectious diseases, including TB. Plant-derived medicines can be produced efficiently and at low cost, and are easily introduced into the pharmaceutical market [3]. The most effective treatment strategy for TB involves identifying multiple therapeutic targets to inhibit pathogen growth using anti-TB medications [3, 9]. The genus *Datura* includes nightshades and various crops employed in agriculture. The species most commonly used in medicine are *Datura stramonium*, *Datura inoxia*, *Datura metel*, *Datura ferox*, and *Datura ceratocaula*. Species of this genus grow in the wild and have been introduced into Asia, Europe, and the Americas [10]. Some species are also found in Africa and Australia. The medicinal and recreational use of *Datura* dates back thousands of years. Commonly known as thorn apple, prickly burr, jimson weed, moon flower, devil’s weed, devil’s cucumber, and trumpet, this plant is found in almost all parts of the world. *Datura* species are rich in tropane alkaloids, mainly in their seeds and flowers, among which scopolamine, hyoscyamine, and atropine are noteworthy. It is indigenous to China, Mexico, Asia, the Caribbean, and the US. *D. innoxia* belongs to the Solanaceae family and possesses significant ethnopharmacological properties [11, 12]. *D. innoxia* has been noted for its diverse therapeutic properties, including antidiabetic, anti-asthmatic, antimicrobial, anti-inflammatory, anticancer, analgesic, cytotoxic, antioxidant, and neurological, insecticidal, and wound-healing effects. However, research on its antimycobacterial activity remains limited [13, 14].

By assessing the anti-mycobacterial properties of plant extracts from *D. innoxia*, the current study fills this knowledge gap. The antibacterial activity of medicinal plant extracts against *Mtb* is essential for drug discovery. To identify natural agents that target different *Mtb* proteins, a variety of in silico techniques have been used, including structure-based virtual screening, pharmacophore modeling, molecular docking, molecular dynamics simulations, and analyses of absorption, distribution, metabolism, excretion, and toxicity (ADMET). The screening and identification of *D. innoxia* extracts with possible therapeutic advantages are greatly aided by this technique.

## Methodology

### In vitro studies

#### Growth conditions

Frozen stocks of H37Ra developed from the single colonies (isogenic) was grown in 30mL Nalgene inkwell bottles containing 10mL of Middlebrook 7H9 broth supplemented with 0.2% (v/v) glycerol (Sigma Chemical Co.), 10% (v/v) oleic acid, albumin, dextrose, catalase (OADC; Difco), and 0.05% (v/v) tyloxapol (Sigma). Cultures were incubated at 37 °C under shaking conditions at 150 rpm in a shaking incubator (New Brunswick Scientific, USA) until the culture reached an optical density (0D6_00_) of 0.5–0.6. MBC were determined on the Middlebrook 7H10 agar supplemented with 0.2% glycerol and 10% OADC.

#### Inoculum preparation

The above H37Ra grown cultures were further diluted into fresh complete 7H9 media to an OD_600_ of 0.001 (to reduce the glycerol content) and further grown to an OD_600_ of 0.2 to 0.3. H37Ra culture was diluted to an OD_600_ of 0.02 and inoculated into the drug containing 96 well plates at a final OD_600_ approximated to 0.01.

#### Antitubercular activity

Fisetin’s antitubercular action was assessed using an MTT assay and the procedure outlined by Martin et al. [15]. with necessary modification. Stock solution (4 mg/mL) of fisetin was prepared in DMSO for minimum inhibitory concentration (MIC) determination. The test compound, isoniazid (positive control) and 0.5% DMSO (negative control) were two-fold serially diluted in 96-well round bottom plates in 100 µL Middlebrook 7H9 media. Afterwards, 100 µL H37Ra culture was dispensed into the wells of 96 well plate with the final cell density approximated to an OD6_00_ of 0.01. The cell density of the *Mtb* was verified by plating the serial dilution of the inoculum in triplicate onto 7H10 agar plates. Agar plates were incubated for 3 weeks for colony forming units (CFUs) enumeration. Microtiter plates were incubated for one week before MICs were read first by an unaided eye. Afterwards, MICs were by confirmed by MTT assay also. 10 µL of 5 mg/mL MTT dye (prepared in PBS) was added to each well. Plates were further incubated for overnight. Next morning, violet formazan precipitation was observed and 50 µL of a solubilization buffer solution, consisting of 20% SDS and 50% DMF, was added to dissolve the formazan [16]. Plates were further incubated for at least 3 h before MICs were determined by visual observation. The presence of bacterial growth was indicated by a change in color from yellow to violet [17]. Minimum bactericidal concentration (MBC) of the compounds were determined by plating the serial dilutions of the concentrations above MIC readouts onto Middlebrook 7H10 agar plates. Agar plates were incubated for 3 weeks under non-shaking conditions and CFUs were counted to record the MBCs. MBC was defined as the lowest concentration that kills 99.9% of the bacilli of the initial inoculum.

### In silico analysis

#### Evaluation of drug profile

The SwissADME server (accessed on September 20, 2024) (SwissADME), which assesses chemicals in terms of absorption, distribution, metabolism, and excretion, was used to analyse the pharmacokinetic and ADMET properties. The SwissADME platform, which was accessible on September 20, 2024, was used to forecast the ADME characteristics of the compounds for pharmacokinetic evaluation. The drug-likeness of the compounds was evaluated using Lipinski’s Rule of Five (RO5). ADME analysis was carried out after entering the SMILES string for fisetin [18]. While StopTox (https://stoptox.mml.unc.edu/) [19] was used for the toxicity studies. pkCSM (http://biosig.unimelb.edu.au/pkcsm/) [20] was employed for detailed pharmacokinetic and toxicity predictions, including absorption, distribution, metabolism, excretion, and toxicity (ADMET) parameters [21].

#### Target protein retrieval

In this study, eight key anti-tuberculosis targets were selected based on their critical roles in cell-wall biosynthesis, fatty acid metabolism, amino acid metabolism, and signal transduction pathways that are critical for *Mtb* growth, survival, and dormancy. These include: Pantothenate kinase (PanK, type 1), Decaprenylphosphoryl-β‐d‐ribofuranose oxidoreductase (DprE1), Enoyl-CoA hydratase 6 (EchA6), Protein kinase B (PknB), Protein kinase A (PknA), Enyol-ACP reductase (InhA), and Aspartate aminotransferase (aspAT). The Protein Data Bank (RCSB PDB) provided the crystal structures of these proteins (PDB IDs: 1UZN, 4BFZ, 4P8C, 5AGS, 5DUF, 5U94, 6B2Q, 6R9W, and 6U7A) in PDB format (accessed on September 20, 2024) (RCSB PDB: Homepage) [22–31].

#### Retrieval and preparation of ligands

Fisetin was chosen as the ligand, and its 2D and 3D structures were obtained from PubChem, created using ChemDraw (Version 21.0), and visualized through Discovery Studio Visualizer (v21.1.0.20298). The structural data file (SDF) format of the NCBI PubChem database (https://pubchem.ncbi.nlm.nih.gov/ ) was used to obtain the structure of each co-crystalized ligand and reference drug isoniazid (INH). For ligand preparation, structural optimization and conformer generation were performed using LigPrep available in Schrödinger’s 2020-3 software (Maestro Wizard version 21.3). Low-energy 3D conformations with correct chiralities were generated and optimized with the OPLS3 force field at a physiological pH of 7.2 ± 0.2, yielding 32 conformers per ligand [32, 33].

#### Protein preparation

Proteins were processed using the Schrödinger suite (Maestro Wizard version 21.3) through the Protein Preparation Wizard. PDB protein structures were imported, and any structural inaccuracies, such as missing residues, were corrected. Missing loops and side chains were reconstructed using the Prime tool, and a heteroatom state was generated using the Epik module (pH = 7.0 ± 2), while metals were treated as zero-order states. Hydrogen atoms were added, hydrogen bonding was optimized, water molecules were removed, disulfide bonds were formed, and the structure was minimized using the OPLS3 force field. Grid files were created to specify the receptor-binding sites, and the van der Waals (vdW) radius scaling was set at 1.0 [34].

#### Protein-ligand docking

Ligand-protein docking was performed using the Schrödinger suite (Maestro Wizard version 21.3). A receptor grid was built to define the size and direction of the binding site for ligand docking. A scoring grid based on the protein crystal structure was generated using the Schrödinger suite (Maestro Wizard version 21.3). Glide in Schrödinger Maestro 21.3 was used for molecular docking using extra precision (XP) modes, allowing customizable ligand sampling. The partial charge cut-off for the ligand atoms was fixed at 0.15, and the van der Waals radius scaling was set at 0.80 [34].

#### Visualization of protein-ligand complexes

Discovery Studio Visualizer (v21.1.0.20298) was used to investigate protein-ligand complexes and identify important interactions. Additionally, PyMOL (Version 2.4.0) was used to further analyze and visualize the docked complexes [33, 35].

#### Density functional theory (DFT) analysis

GaussView (version 5.0.8) was used to construct 3D structures of Fisetin, and Gaussian 09 W was used to optimize them. The physicochemical properties of the selected compounds were examined using density functional theory (DFT). CPCM solvent models were used for the computations using the B3LYP approach. The bandgap (ΔEGap = ELUMO – EHOMO) was computed as the difference between the highest occupied molecular orbital (HOMO), lowest unoccupied molecular orbital (LUMO), and the energy gap between them. Additionally, DFT studies have shed light on the molecular electrostatic potential (MEP) of compounds [36].

#### Molecular dynamics (MD) simulations

The MD trajectory was analyzed using AMBER 20 software. Three simulation steps were performed for the system setup and analysis [37]. In the trajectory analysis step, five different trajectories were generated: hydrogen bond analysis (H-bond analysis), radius of gyration (RoG), solvent-accessible surface area (SASA), root mean square deviation (RMSD), and root mean square variation (RMSF). While RMSD measures the time-dependent fluctuations of the protein structure, RMSF measures residue-wise fluctuations. Furthermore, the compactness and relaxation of the complexes were assessed using Rg, as structural compactness is essential for preserving complex stability. The amount of protein surface accessible to the solvent was ascertained by SASA analysis, which measured the distribution of hydrophilic and hydrophobic residues. Additionally, a script was written to perform principal component analysis and dynamic cross-correlation (DCCM) using the Bio3D package in R [38–40]. Atomic coordinates were used to project the first two principal components (PC1 and PC2). The eigenvalues and eigenvectors were obtained by diagonalizing this matrix [41].

#### Free energy binding MMGBSA computations

Using MD shots, binding free energy was computed to learn more about the energetic values and structure of the complexes. This was performed using the MMPBSA/MMGBSA modules in conjunction with Amber20. The total energies of fisetin and the reference drug (INH) in complex with 5U94 were computed using the last two nanoseconds of data from 1000 snapshots of both complexes [42, 43].

## Results

### Antitubercular activity

The results showed the MIC and MBC of the phytocompound against *Mtb* H37Ra. Phytocompound Fisetin exhibited a MIC value of 100 µg/mL and MBC value of 200 µg/mL, suggesting that the compound has a bacteriostatic and bactericidal effect (Supplementary Table S1). This phytocompound is a promising candidate for further development as an antitubercular agent.

### Evaluation of drug profile

The ADMET study of fisetin, an important bioactive compound from *D. innoxia*, was performed to determine its potential as an anti-mycobacterial agent against TB. The five-rule evaluation of fisetin offers an appealing profile in terms of drug-like characteristics. We found an MW of 286.24 g/mol, and in every instance, no significant target was absent. With six HB acceptors and four HB donors, the molecule is ideal for hydrogen bonding. The absorption score was high because of a high TPSA of 111.13 Å², which indicates significant GI absorbance, and the polar refractivity was 76.01. With an average logP value of 1.55 when integrating estimates from many models—iLOGP 1.5, XLOGP3 1.97, WLOGP 2.28, MLOGP − 0.03, and Silicos-IT LogP of 2.03—lipophilicity was moderate. These characteristics suggest that this chemical has a moderate level of lipophilicity, which is useful for passing through the lipid cell membranes.

ESOL Log S of -3.35 and AliLog S of -3.93 were used to predict the solubility properties reasonably. Finally, log Kp was used to quantify the permeability of human skin.

Fisetin is a substrate for CYP2D6 and CYP3A4, according to studies, suggesting that these cytochrome P450 enzymes are in charge of fisetin metabolism. Additionally, it inhibits CYP1A2 but has no effect on CYP2C19 or CYP2C9, which lowers the likelihood of drug interactions with these enzymes. Because fisetin is not a substrate of P-glycoprotein, there may be less efflux of this substance, which could lead to increased cellular accumulation.

**Fig. 1:**
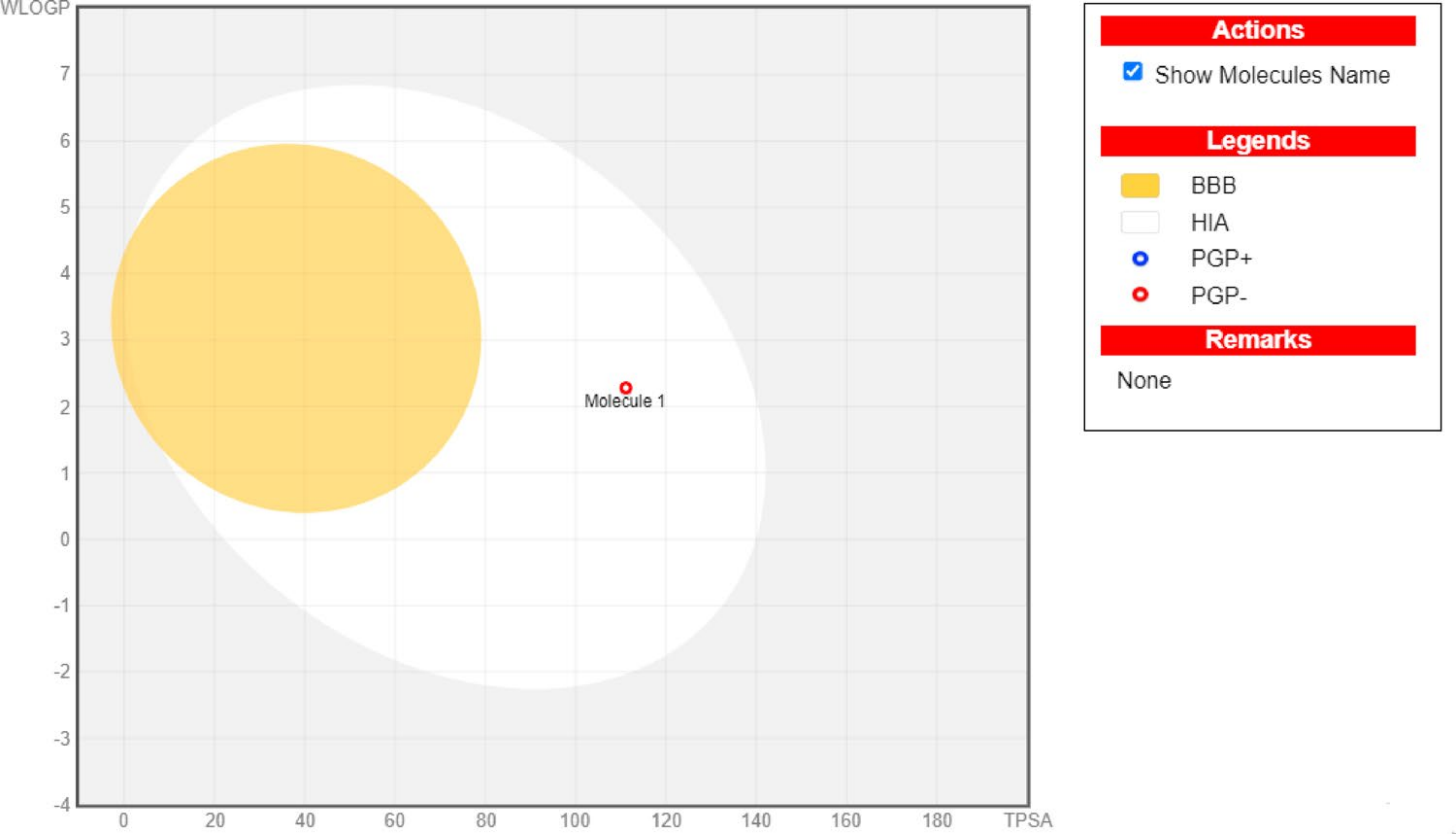
The BOILED-Egg model shows the distribution and absorption characteristics of fisetin. The yellow region, which resembles an egg yolk, indicates the penetration of the blood-brain barrier (BBB), and the white zone denotes absorption in the gastrointestinal system. Compounds are colored blue when they are P-glycoprotein substrates (PGP+) and red when they are non-substrates (PGP-)

Its bioavailability was determined to be 0.55, indicating significant systemic distribution and therapeutic effects despite its incapacity to cross the blood–brain barrier (Fig. 1). The molecular structure showed a Brenk warning (catechol) and PAINS alert (catechol), indicating certain characteristics that should be considered. Fisetin can be regarded as relatively straightforward to synthesize, with a synthetic accessibility score of 3.16 (Table 1), and more research is necessary.

**Table 1:** ADME analysis of Fisetin, a bioactive compound from *D. innoxia*

ADMET Properties	Fisetin
MW	286.24
#H-bond acceptors	6
#H-bond donors	4
TPSA	111.13
iLOGP	1.5
XLOGP3	1.97
WLOGP	2.28
MLOGP	-0.03
Silicos-IT Log P	2.03
Consensus Log P	1.55
ESOL Log S	-3.35
Ali Log S	-3.93
Log Kp (cm/s) skin permeation	-6.65
CYP2D6 substrate	Yes
CYP3A4 substrate	Yes
CYP1A2 inhibitior	Yes
CYP2C19, 2C9 inhibitior	No
GI absorption	High
BBB permeant	No
P-gp substrate	No
Bioavailability Score	0.55
Lipinski	0 violation
PAINS #alerts	1 catechol_A
Brenk #alerts	1 catechol
Leadlikeness	Yes
Synthetic Accessibility	3.16

**Fig. 2:**
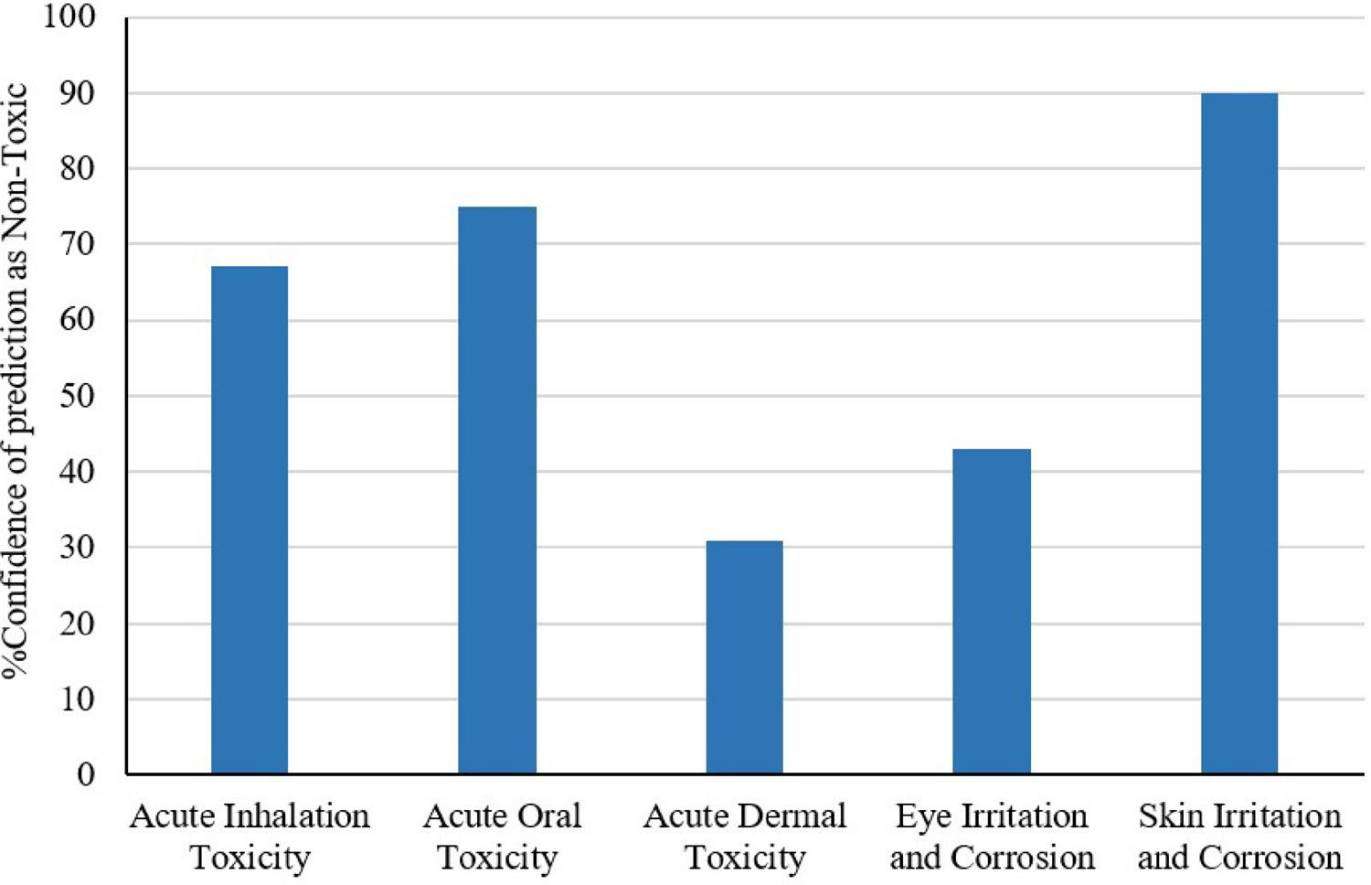
Toxicity prediction of fisetin using online tool stoptox

Fisetin showed notable non-toxic behavior against acute inhalation, oral and dermal routes, skin irritation, and corrosion, according to the Stoptox toxicity investigation. However, there was only a small percentage of certainty that it was non-toxic for dermal (Fig. 2). The pkCSM tool offers more accurate pharmacokinetic and toxicity predictions, including absorption, distribution, metabolism, excretion, and toxicity (ADMET) characteristics. Supplementary Table S2 presents these findings.

### Molecular docking

**Fig. 3:**
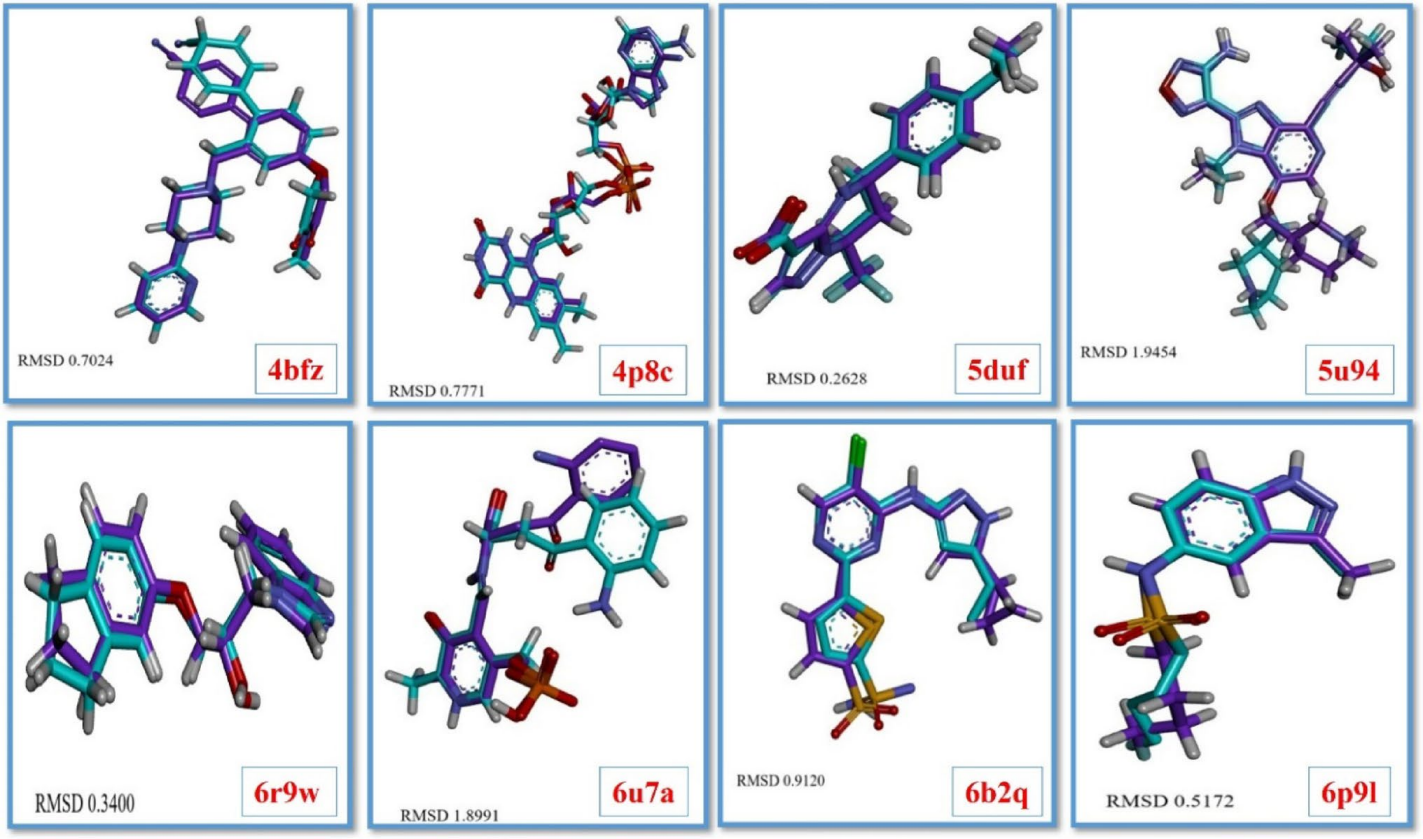
RMSD value of superimposed experimental pose (purple) and re-docked pose (cyan) of CCL for 4BFZ, 4P8C, 5DUF, 5U94, 6B2Q, 6P91, 6R9W, and 6U7A targets

Fisetin’s anti-mycobacterial qualities led to its selection for in silico investigation. A number of targeted *Mtb* proteins, such as β-ketoacyl acyl carrier protein synthase I (KasA), Pantothenate kinase (PanK, type 1), Decaprenylphosphoryl-β-d-ribofuranose oxidoreductase (DprE1), Enoyl-CoA hydratase 6 (EchA6), Protein kinase B (PknB), Protein kinase A (PknA), Enoyl-ACP-reductase (InhA), and Aspartate aminotransferase (AspAT), were investigated in order to better understand the compound’s binding potential. The PDB IDs for these proteins are 4BFZ, 4P8C, 5AGS, 5DUF, 5U94, 6B2Q, 6R9W, and 6U7A are the corresponding PDB IDs for these proteins. By analyzing the binding affinities of fisetin for the target proteins, we determined the binding affinities for the respective pairs. To confirm the efficacy of the docking study, the CCLs for the respective proteins were re-docked into the binding pockets. The docking study was considered valid if the RMSD values for the re-docked CCLs were below 2 Å and the docked structures were comprised of the stacked structure (Fig. 3). Supplementary Table S3 contains information about the ligand names, coordinates, and box sizes for the corresponding redocked complexes. Several interesting interactions were observed in the docking investigation of fisetin against numerous *Mtb* protein targets (Table 2). With a docking score of −10.8, the fisetin complex with protein kinase B (PknB) (PDB ID: 5U94) obtained the highest score. This implies that fisetin has a substantial interaction with this vital enzyme, making it a crucial target for TB.

**Table 2:** Docking scores of Fisetin with selected targeted *Mtb* proteins

Serial no.	Mtb protein target	PDB ID	Docking scores
1	Pantothenate kinase (PanK, type 1)	4BFZ	−7.593
2	Decaprenylphosphoryl-β‐d‐ribofuranose oxidoreductase (DprE1)	4P8C	−8.734
3	Enoyl-CoA hydratase 6 (EchA6)	5DUF	−7.772
4	Protein kinase B (PknB)	5U94	−10.817
5	Protein kinase A (PknA)	6B2Q	−7.81
6	β-ketoacyl acyl carrier protein synthase I (KasA)	6P91	−6.506
7	Enyol-ACP-reductase, (InhA)	6R9W	−9.325
8	Aspartate aminotransferase (aspAT)	6U7A	−9.793

Similarly, Aspartate aminotransferase (AspAT) (PDB ID: 6U7A) and Enyol-ACP-reductase, (InhA) (PDB ID: 6R9W) demonstrated considerable binding affinities, with docking scores of −9.7 and − 9.3, respectively (Table 2). These three targets were selected for further docking interaction analysis because Fiestin showed the highest binding scores with them and a comparative study was performed using the CCL of each target along with the FDA-approved drug INH as the reference. The two-dimensional and three-dimensional structures, along with the SMILE representation of fisetin, are presented in Table 3. These findings indicate that fisetin could serve as an inhibitor of several crucial *Mtb* proteins.

**Table 3:** 2D, 3D Structures and SMILE of Fisetin

Name of the compound	2D structure	3D structure	SMILE
Fisetin			C1=CC(=C(C=C1C2=C(C(=O)C3=C(O2)C=C(C=C3)O)O)O)O

**Fig. 4:**
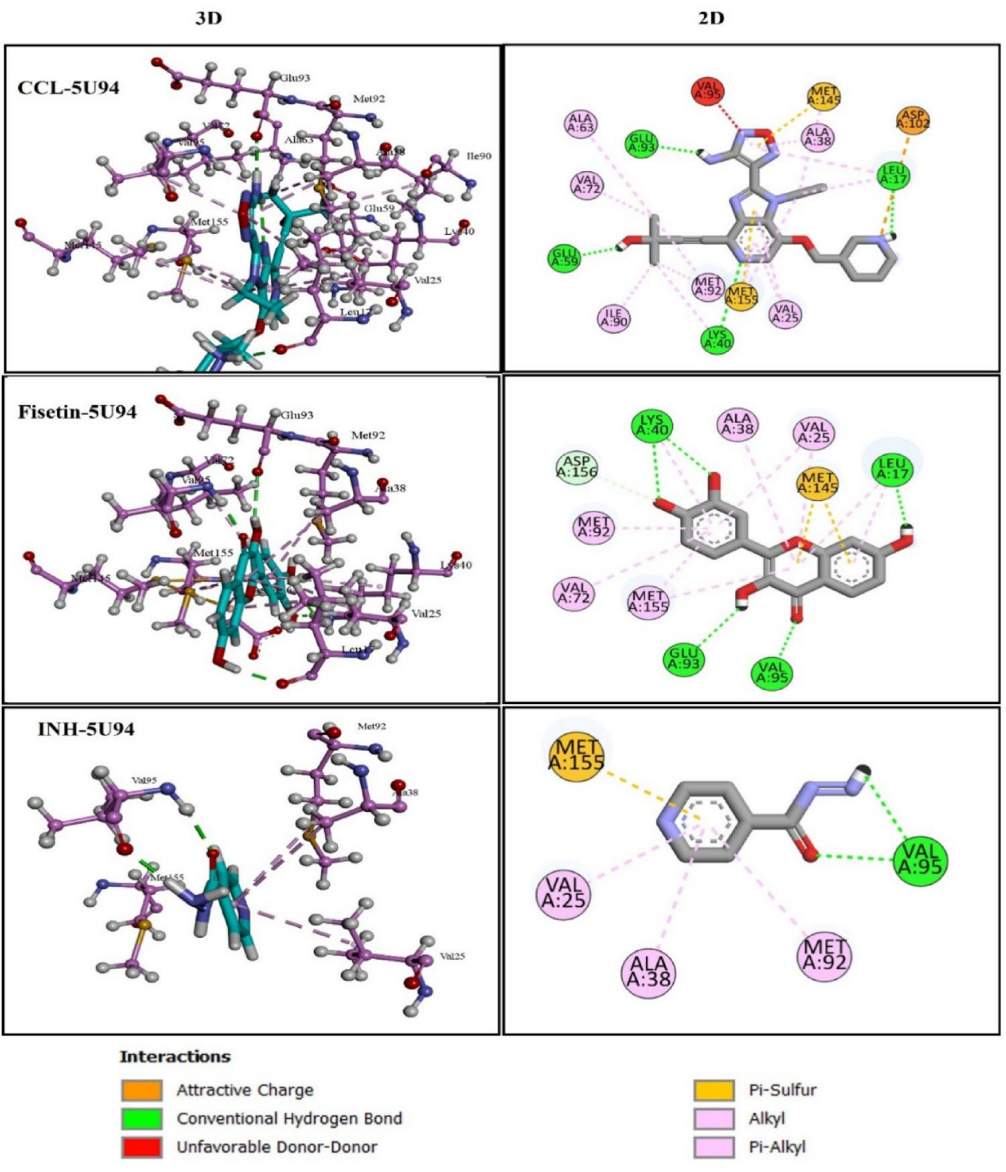
2D and 3D visualization of docked complexes of Protein 5U94 with CCL, fisetin and INH showing Ligand-Residue Interactions

According to the docking scores, Fisetin (−10.87 kcal/mol) is strongly interacting with Protein kinase B (PknB) (PDB ID: 5U94), as compared to CCL (−8.06 Kcal/mol) and INH (−5.47 Kcal/mol) (Table 4). Consequently, Discovery Studio was used to visualize the two- and three-dimensional interactions of these CCL with 5U94 target protein to explore binding site residues. It create H-bond interactions with residues Lys40, Glu93, Glu59 and Leu17 at distance less than 3 Å. Residues Ala63, Leu17, Met145, Met155, Val72, Met92, Lys40, Ile90, Val25, Ala38, VAl95 surrounded the CCL by hydrophobic contacts (Fig. 4). Fisetin also formed interaction same way as CCL and involved to form H-bond contact with residues Lys40, Val95, Glu93 and Leu17 with distance of H-bonds are les then 2.6 Å. It involved same residues for hydrophobic contact as for CCL (Fig. 4). While FDA approved reference drug showed less prominent interactions as it only formed single H-bond with residue Val95 and less numbers of residues are involved in hydrophobic interactions. So fisetin could formed a strong interaction at the binding site of 5U94 and could act as a Potential Inhibitor of *Mtb*.

**Table 4:** H-bond and hydrophobic interactions of CCL (G93), Fisetin and INH with 5U94

Mtb Protein Target: Protein kinase B (PknB) PDB ID:5U94				
Ligands	Docking score (Kcal/mol)	H-Bonds	Distance (Å)	Hydrophic contacts
CCL (G93)	−8.06	Lys40, Glu93, Glu59, Leu17,	2.77766 2.12968 1.77801 2.15033	Ala63, Leu17, Met145, Met155, Val72, Met92, Lys40, Ile90, Val25, Ala38, VAl95
Fisetin	−10.87	Lys40, Val95, Glu93, Leu17	2.36271 1.99841 2.60027 1.95442	Leu17, Val25, Ala38, Met155, Leu17, Val25, Lys40, Val72, Met92,
INH	−5.47	Val95	2.01175	Val25, Val38, Met92

**Fig. 5:**
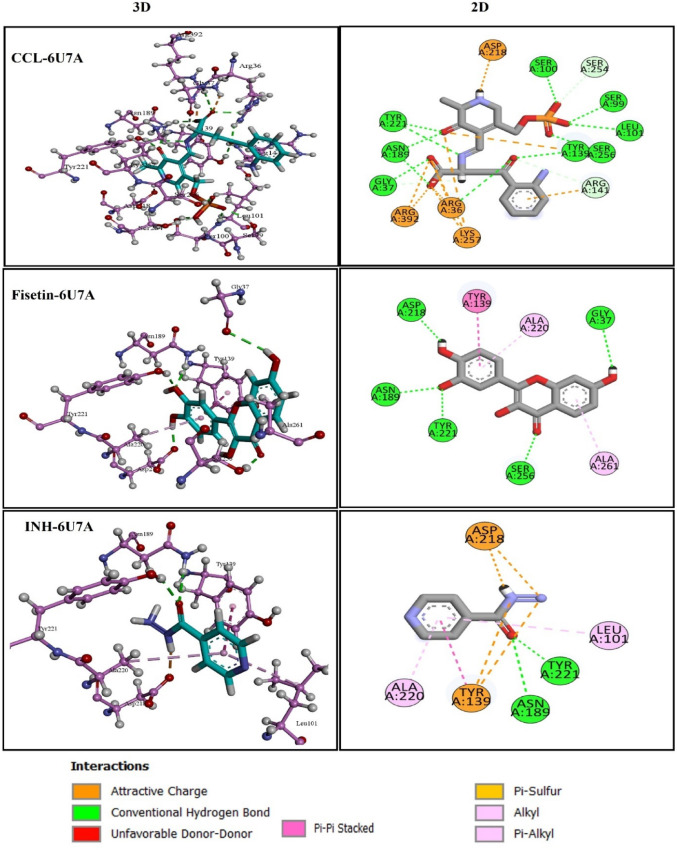
2D and 3D visualization of docked complexes of Protein 6U7A with CCL, fisetin and INH showing Ligand-Residue Interactions

The two-dimensional and three-dimensional interactions of CCL with the target 6U7A indicated that the compound formed conventional hydrogen bonds with residues Arg36, Gly37, Ser99, Ser100, Leu101, Tyr139, Asn189, Tyr221, and Ser256. But no hydrophobic interactions were observed. While Fisetin with comparable docking score with CCL (Table 5), formed H-bonds with Asn189, Tyr221, Ser256, Gly37 and Asp218. Additionally, pi-pi stacked bonding with Tyr139 and alkyl bonding with Ala261 and Ala220, added more stability to Fisetin-6U7A complex (Fig. 5; Table 5). In comparison, INH showed less docking score − 3.419 Kcal/mol, formed H-bonds with two residues Asn189 and Tyr221 and pi-alkyl interactions with Tyr139, Leu101 and Ala220 (Fig. 5).

**Table 5:** H-bond and hydrophobic interactions of CCL (QOP), Fisetin and INH with 6U7A

Aspartate aminotransferase (aspAT) PDB ID:6U7A				
Ligands	Docking score (Kcal/mol)	H-Bonds	Distance (Å)	Hydrophic contacts
CCL (QOP)	−15.25	Arg36, Gly37, Ser99, Ser100, Leu101, Tyr139, Asn189, Tyr221, Ser256,	2.10791 2.55341 1.93952 1.9317 2.10113 2.09416 2.1527 3.04743 1.79167	–
Fisetin	−9.793	Asn189, Tyr221, Ser256, Gly37, Asp218,	2.01251 2.06951 1.93908 2.54656 1.78089	Tyr139, Ala261, Ala220,
INH	−3.419	Asn189, Tyr221	2.37938 2.08976	Tyr139, Leu101, Ala220

**Fig. 6:**
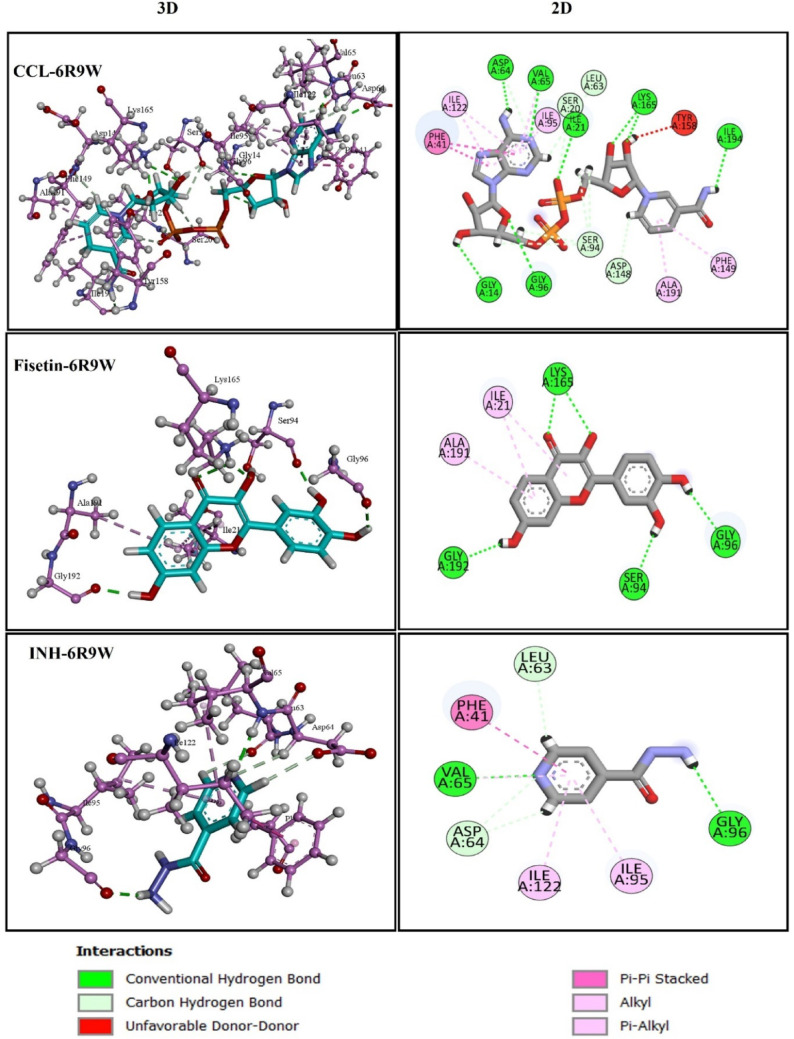
2D and 3D visualization of docked complexes of Protein 6R9W with CCL, fisetin and INH showing Ligand-Residue Interactions

Graphical analysis of CCL with protein 6R9W used to explore the binding site residues features (Fig. 6). It was seen that amino acid residues Ile21, Val65, Gly96, Lys165, Gly14, Asp64, and Ile194 are involved in H-bonding with ligand. While residues Ala91, Phe149, Ile95, Ile122, and Val65 are involved in hydrophobic interactions and residue Phe41 is aromatic in nature, formed pi-pi stacked interaction with ligand.

The two-dimensional and three-dimensional interactions of docked complex of fisetin with target 6R9W indicated that the compound establishes a conventional hydrogen bond with the Lys165, Gly192, Ser94, and Gly96. Pi-alkyl interactions were observed with Ile21, Ala191, making less stable compared to CCL. In contrast, INH formed only two conventional hydrogen bonds with residues Val65, Gly96. Notably, the Phe41 residue participates in pi-pi stacked interactions make it stable, similar to CCL. Moreover, INH also demonstrated Pi-alkyl interactions with Val65, Ile95 and Ile122 (Fig. 6; Table 6).

**Table 6:** H-bond and hydrophobic interactions of CCL (QOP), Fisetin and INH with 6U7A

Mtb Protein Target: Enyol-ACP-reductase, (InhA) PDB ID:6R9W				
Ligands	Docking score (Kcal/mol)	H-Bonds	Distance (Å)	Hydrophic contacts
CCL (NAD)	−14.09	Ile21, Val65, Gly96, Lys165, Gly14, Asp64, Ile194,	2.10534 2.15847 2.66258 2.81113 2.62269 1.9964 2.52793	Phe41, Ala91, Phe149, Ile95, Ile122, Val65
Fisetin	−9.325	Lys165, Gly192, Ser94, Gly96,	2.0646 1.86431 1.77891 2.2472	Ile21, Ala191,
INH	−6.55	Val65, Gly96	2.18799 2.10955	Phe41, Val65, Ile95, Ile122,

### Density functional theory (DFT) calculations

**Fig. 7:**
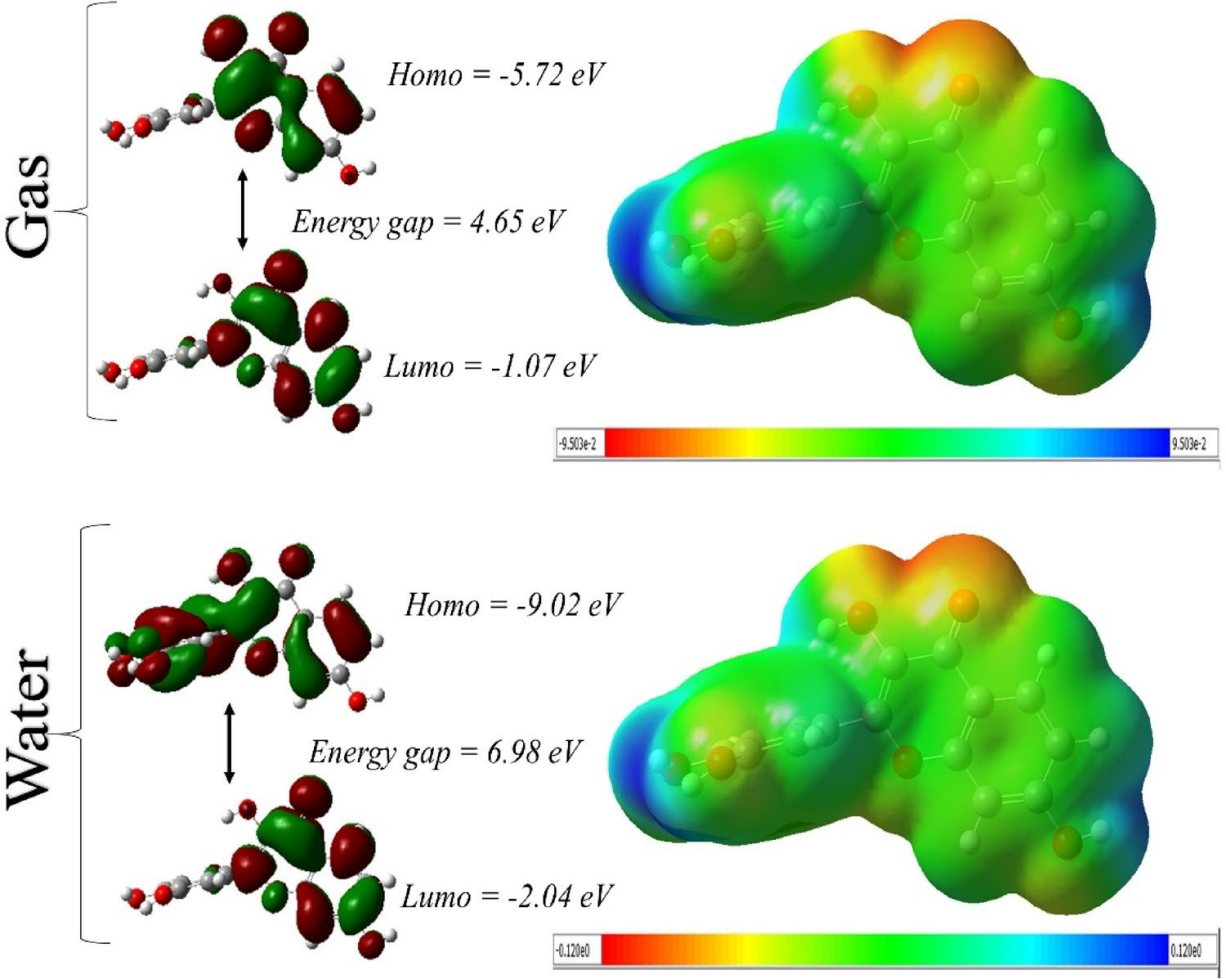
DFT analysis of fisetin representing both solvent and gaseous phases

Fisetin’s electrical characteristics are clearly modulated by the solvent, according to DFT study of the gas and water phases (Table 7; Fig. 7). Fisetin has considerable chemical stability and reactivity in the gas phase with a HOMO energy of − 5.725 eV and a LUMO energy of − 1.074 eV, equating to an energy gap of 4.650 eV. In contrast, solvation in water considerably stabilizes both frontier orbitals, expanding the energy gap to 6.980 eV and moving the HOMO and LUMO energies to − 9.020 and − 2.040 eV, respectively, indicating improved kinetic stability and decreased reactivity in an aquatic environment. Stronger polarization and better solute–solvent interactions are reflected in the dipole moment, which increased from 4.225 Debye in the gas phase to 4.614 Debye in water. Consequently, water has a higher ionization potential and electron affinity, which increase the electronegativity and hardness while decreasing the softness, further supporting the decreased chemical reactivity in the solution. Although the electrophilicity index increases significantly in water (4.382 eV) compared to that in the gas phase (2.485 eV), suggesting a greater propensity to absorb electrons in the aqueous phase, the negative chemical potential values indicate thermodynamic stability in both phases. Overall, these findings show that water electronically stabilizes fisetin, increases its polarity and electrophilic nature, and makes it less reactive than in the gas phase, all of which are in line with its predicted behavior in biological settings.

**Table 7:** Comparative DFT analysis of fisetin in water and gas phases

Properties	Calculated Energies	
	Gas	Water
Homo (eV)	−5.725	−9.020
Lumo (eV)	−1.074	−2.040
Egap = Elumo - Ehomo (eV)	4.650	6.980
Dipole moment (Debye)	4.225	4.614
Ionization potential I= -Ehomo	5.725	9.020
Electron affinity A= -Elumo	1.074	2.040
Electronegativity χ= (I + A)/2	3.399	5.530
Hardness η= (I − A)/2	2.325	3.490
Softness S = 1/2η	0.215	0.143
Electrical potential µ = −χ	−3.399	−5.530
Electrophilicity ω = µ2/2η	2.485	4.382

### Molecular dynamic simulations of 5U94-Fisetin docked complex

**Fig. 8:**
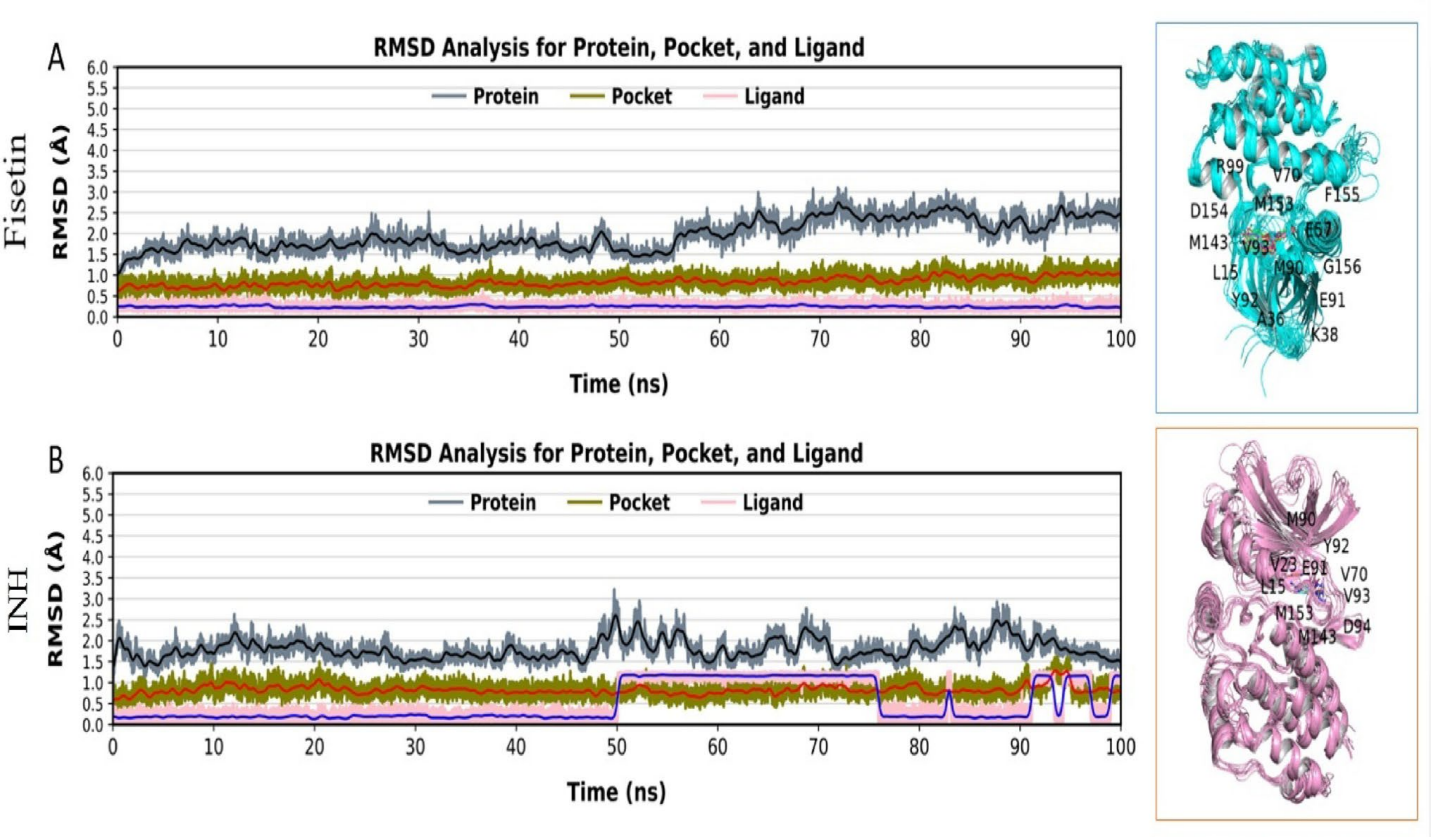
RMSD plots of Fisetin_5U94 (**A**) and INH _5U94 (**B**) over a 100ns MD simulation run

While molecular dynamics (MD) simulations evaluated the structural shape and mobility of the complex in real time, docking analyses demonstrated interactions between the protein and ligand under static settings. Simulations of 100 ns were performed to examine the dynamic behavior of Fisetin and INH with respect to the target protein (PDB ID: 5U94). This made it possible to thoroughly analyze the variations and flexibility of the allosteric site. The root-mean-square deviation (RMSD) of the backbone atoms was computed during the 100 ns MD simulation to assess the stability of the protein-ligand combination [28, 44]. The RMSD analysis demonstrated that the fisetin–protein complex retained good structural stability during the 100 ns MD simulation compared to the reference drug (INH). The protein backbone RMSD in Fig. 8A (fisetin) remained largely stable within ~ 2.0–3.0 Å following an initial equilibration period, indicating a lack of considerable conformational drift. Fisetin was firmly anchored within the active site during the simulation, as seen by the binding pocket RMSD fluctuating slightly at about 1.0–1.5 Å whereas the ligand RMSD continuously stayed below about 1.0 Å. There are discernible variations and brief jumps at the middle and end of the simulation, especially in the ligand RMSD, which indicates sporadic rearrangements within the binding site, even if the protein and pocket RMSD values in Fig. 8B (reference drug) are identical. These observations were supported by the side-view interaction plots, which illustrated the binding of fisetin molecules to the active site residues throughout the simulation run. RMSD plots and active site interactions imply the formation of a stable complex between fisetin molecules and protein 5U94, with a better binding affinity than the standard drug (INH) molecules.

**Fig. 9:**
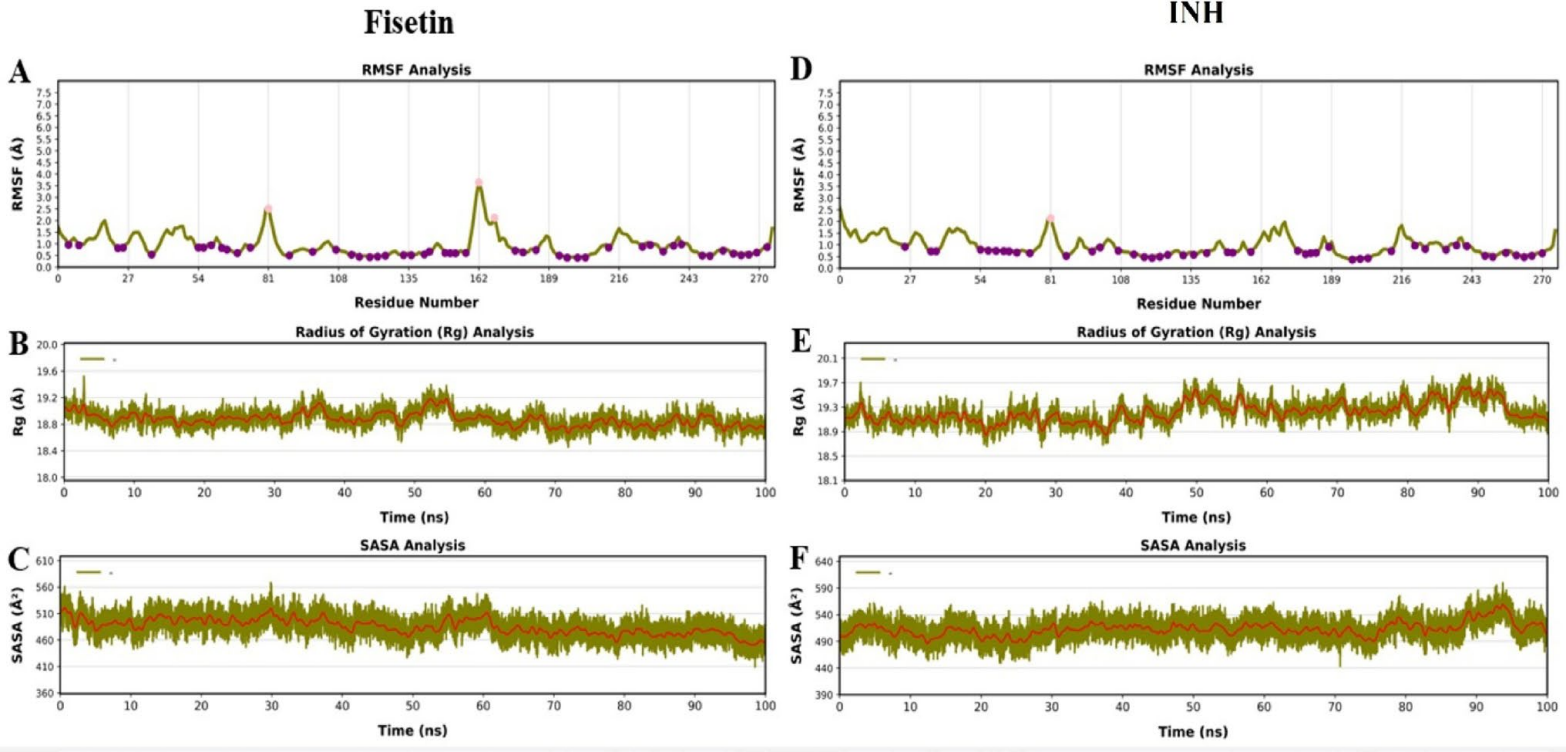
Plots of RMSF, Rg, and SASA for complexes Fisetin_5U94 and INH _5U94; Fisetin_5U94 (**A**-**C**) and INH _5U94 (**D**-**F**)

Further clarity is provided by the RMSF, radius of gyration (Rg), and solvent-accessible surface area (SASA) analyses, where details are revealed of how the protein interacts in the presence of fisetin compared to the INH. Compared to the reference complex, the fisetin-bound complex had higher residue-level B-factors, indicative of higher flexibility at some points, especially in the vicinity of the active site residues. This higher flexibility of the fisetin-binding pocket indicates adaptive conformational flexibility that allows stable binding despite the potential disruption of the overall protein structure. The INH complex had lower RMSF values for the majority of the residues, indicating higher rigidity in the binding mode (Fig. 9A-B).

Although the values in the reference complex are slightly higher and have a larger variation in Rg, with an obvious hint of expansion and laxer packing, the Rg calculations indicate that the fisetin-protein complex remains slightly more compact and stable throughout the 100 ns simulation run, with only the slightest deviations around the mean (Fig. 9C-D). The fisetin complex appears to be more compact in general, with reduced solvent exposure and a more packed protein chain, consistently falling and with a smaller variation in the extent of fluctuations. In comparison, the INH complex had higher and erratically varying SASA values, with increased surface exposure and a less compact structure (Fig. 9E-F). Despite the presence of flexibility in the region around the active site of the protein, the results are clear that fisetin increases the compactness of the protein and is better than the INH at maintaining a dynamically stable and more densely packed complex.

### DCCM analysis

**Fig. 10:**
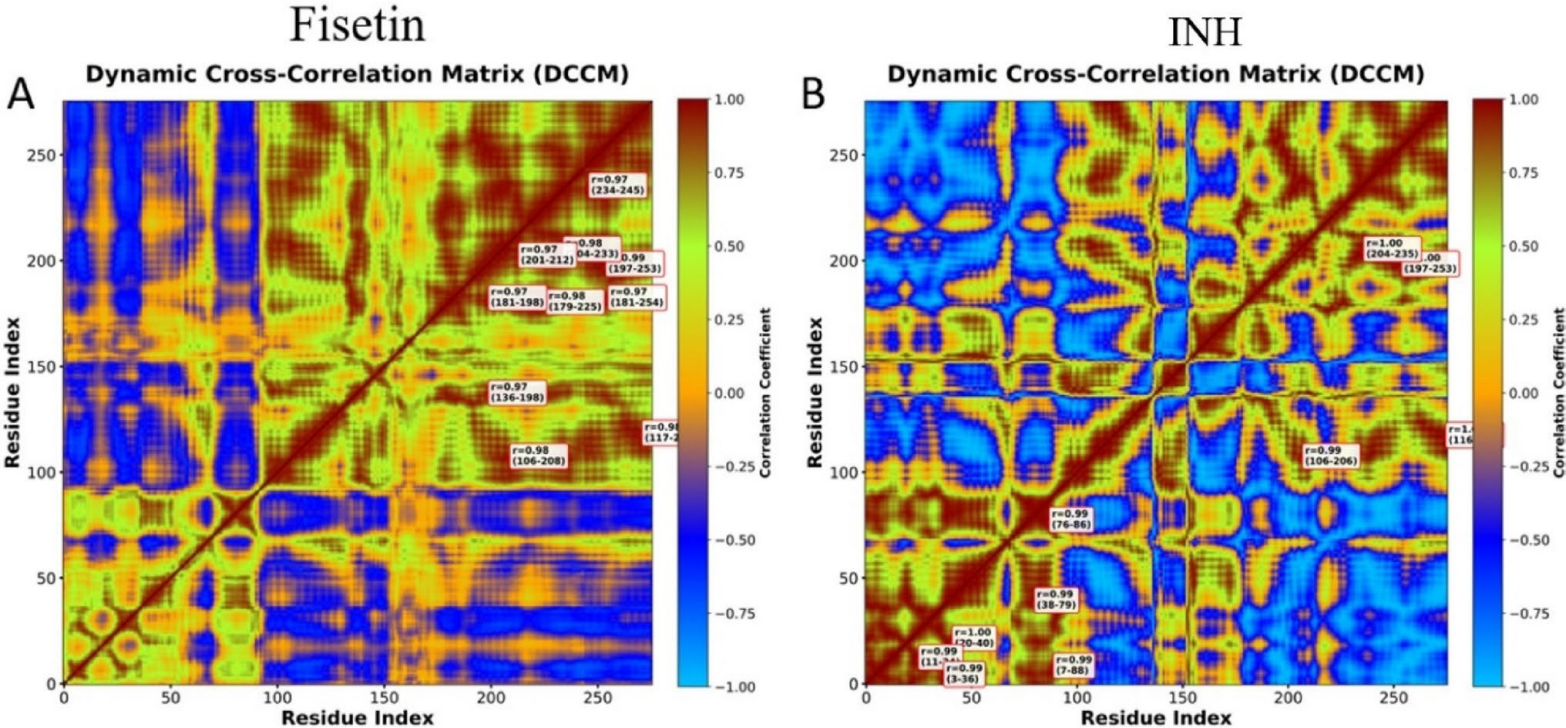
Pots of Fisetin_5U94 (**A**) and INH _5U94 (**B**) complexes showing dynamic cross-correlation matrix

In contrast, the DCCM computation demonstrated clear differences between the motion of the reference drug (INH) and the protein attached to fisetin. Large areas were brightly lit with a high positive correlation (from red to yellow) when the protein was linked to fisetin, suggesting that the regions worked well together in a highly coordinated manner. This implies that the coordinated control of protein dynamics is a function of fisetin (Fig. 10A). The INH -protein complex, on the other hand, has a more intermittent correlation map with some regions of positive correlation and larger blue regions indicating anti-correlated movements. This suggests the uneven and more independent motions of the different domains of the protein, as well as the less coordinated motions of individual protein residues (Fig. 10B). In summary, the DCCM results showed that the fisetin–protein ligand complex has the potential to be dynamically stable and favorable compared to the existing drug.

### PCA and FEL analysis

**Fig. 11:**
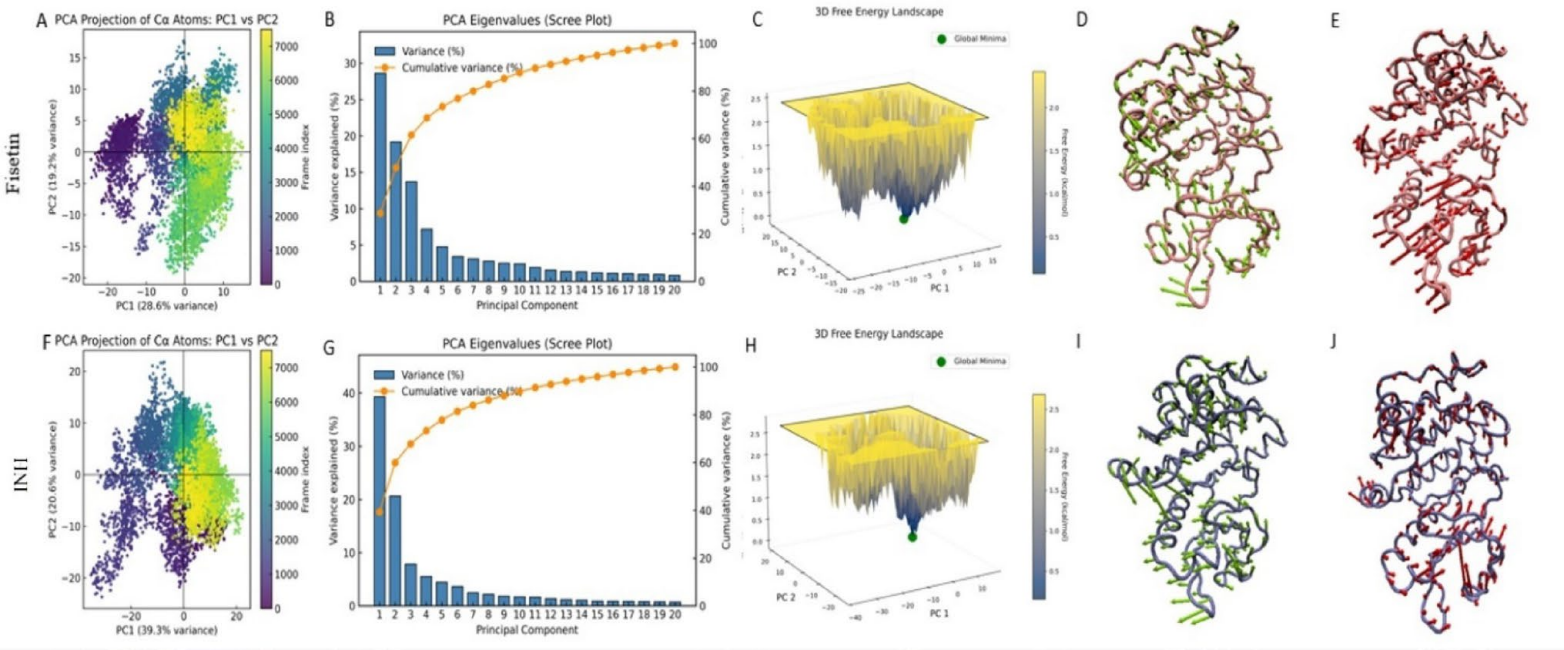
PCA and FEL analysis plots Fisetin_5U94 and INH _5U94 complexes; Fisetin_5U94 (**A**-**E**) and INH _5U94 (**F**-**J**)

Principal component analysis (PCA) and free energy landscape (FEL) data showed that the dynamic behavior of the fisetin-bound complex differed from that of the INH. The fisetin the Cα atoms of the fisetin system revealed a broader and more ordered distribution along PC1 and PC2, with PC1 contributing approximately 28.6% and 19.2% of the total variance, respectively. This implies that the first few primary components contain the majority of the motions. This finding is supported by the corresponding scree plot, which shows that ordered and collective conformational dynamics as the initial components significantly contributed to the cumulative variance. Throughout the simulation, the fisetin complex FEL continuously displayed a deep, well-defined global minimum with a relatively small basin, indicating convergence to a stable, energetically beneficial structural state (Fig. 11A-E). The INH complex, on the other hand, shows a larger contribution from PC1 (~ 39.3%), but it also has a wider free energy basin and a more dispersed PCA distribution, which are signs of more conformational variability and less constrained mobility (Fig. 11F-J). Compared to the reference drug (INH), fisetin promoted more coherent and energetically stable conformational dynamics in the protein, facilitating the establishment of a dynamically well-converged and stable protein–ligand complex, according to PCA and FEL studies.

### Energy component from MMPBSA and energy decomposition per residues analysis

**Fig. 12:**
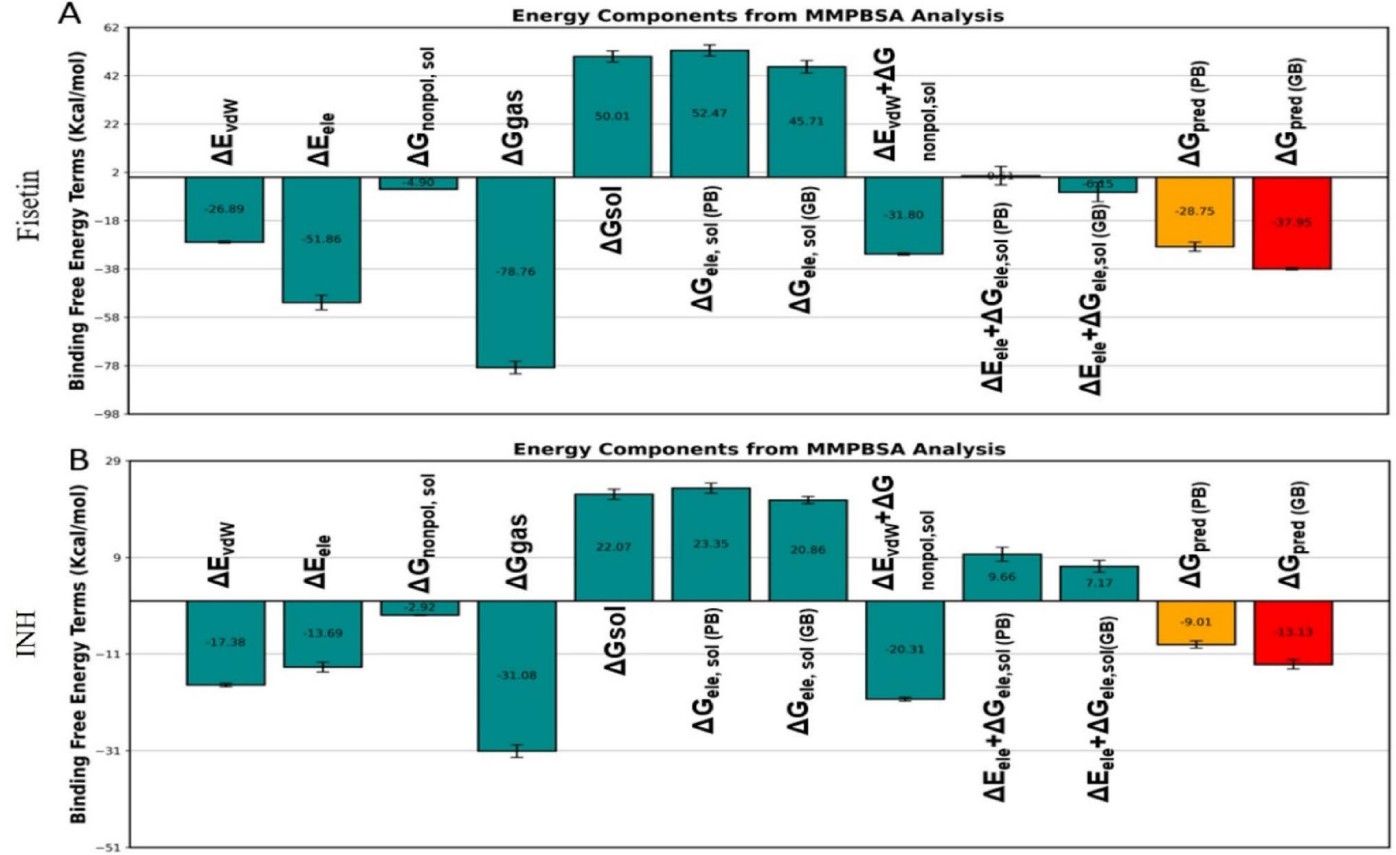
Energy component plots of Fisetin_5U94 (**A**) and INH _5U94 (**B**) complexes from MMPBSA analysis

According to the MMPBSA analysis, fisetin has a more favorable total binding free energy (ΔG_bind) and a stronger binding affinity toward the target protein than the INH. Van der Waals (ΔE_vdw) and electrostatic (ΔE_ele) interactions were the main drivers of fisetin binding, with a highly favorable gas-phase energy (ΔG_gas) that surpassed the unfavorable polar solvation energy (ΔG_sol). The complex was further stabilized by the nonpolar solvation contribution, leading to an overall negative ΔG_bind (-37.95 Kcal/mol). In contrast, the reference drug exhibited weaker gas-phase interactions and a less favorable balance between electrostatic and solvation terms, resulting in a lower binding strength than that of fisetin (Fig. 12).

**Fig. 13:**
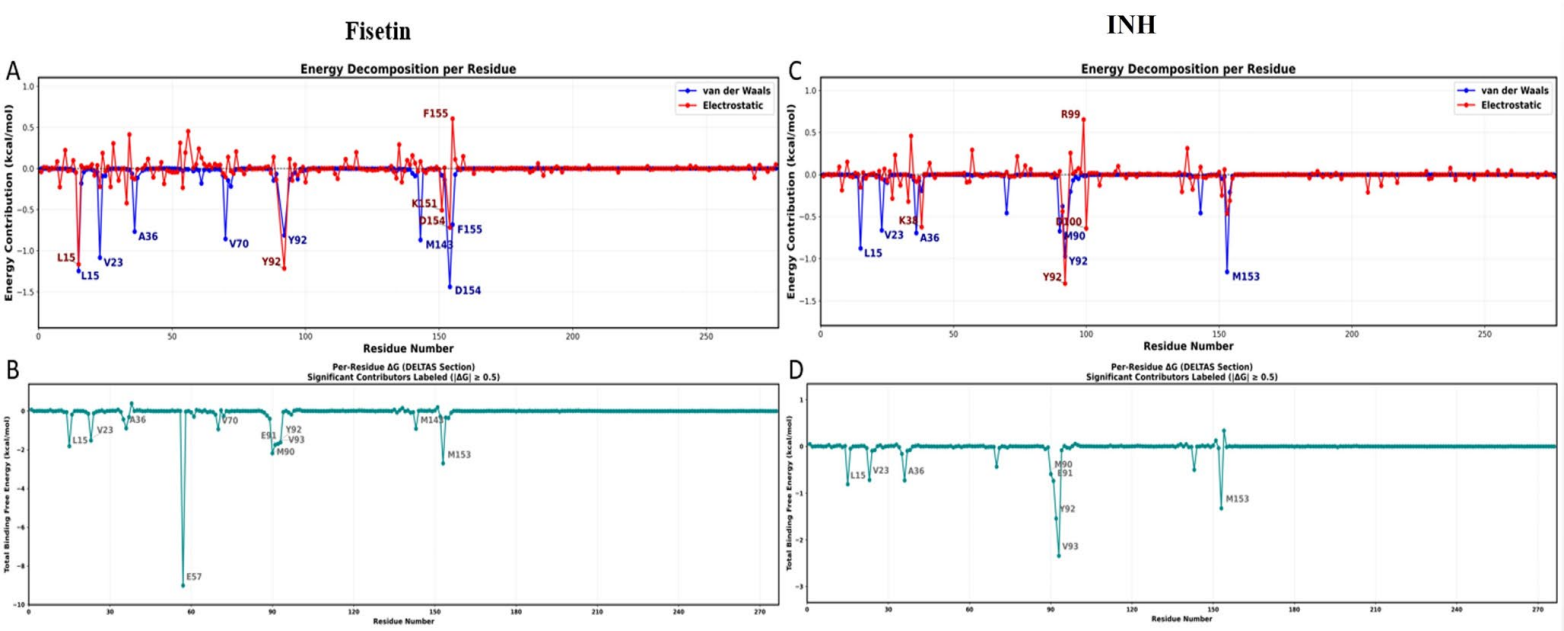
Energy decomposition per residues plots Fisetin_5U94 (**A**) and INH _5U94 (**B**) complexes

Fisetin binding is stabilized by important residues inside the active site, according to per-residue energy breakdown (Fig. 13). Several amino acids contribute significantly to strong van der Waals and electrostatic interactions, revealing a well-anchored binding mechanism. Less-than-ideal packing and weaker residue-level stabilization are suggested by the reference drug’s interactions with overlapping residues, but with less significant contributions. Overall, the better binding stability and interaction profile of fisetin compared to that of the reference medication during the MD simulation are supported by the combined energy component and residue breakdown analyses.

## Discussion

TB is one of the oldest and most widespread diseases in humans. This bacterial infection is caused by *Mtb*. TB is ranked as leading cause of death from a single micro-organism [45]. In recent years, there has been growing interest in medicines derived from natural products of plant origin. Plants are a significant source of biologically active secondary metabolites, which have substantial therapeutic potential. The World Health Organization estimates that approximately 80% of people in developing and underdeveloped nations rely on traditional herbal or botanical remedies for their primary healthcare needs [46]. Natural compounds have been shown to be effective templates for creating novel pharmacological scaffolds and have garnered significant attention as potential medicines against TB [47]. Several reviews have examined antitubercular compounds derived from natural sources [48]. Several notable recent examples of natural product molecules that might be appealing candidates for creating medications to treat TB have been highlighted in a study [49]. This study explored the anti-tuberculosis effects of phytocompound Fisetin (Sigma-Aldrich). Anti-tuberculosis compounds have been obtained from a number of medicinal herbs [50]. In contrast, *D. innoxia*, popularly known as Thorn Apple, Downy Thorn Apple, or Indian Apple, has been related to antibacterial, antioxidant, anticancer, and phytochemical properties, among others [51–54].

This study evaluated the antitubercular properties of fisetin using in vitro techniques. Fisetin’s effectiveness as an antibacterial against *Mtb* H37Ra was established through its MIC and MBC values of 100 µg/mL and 200 µg/mL, respectively. These findings indicate that such a compound may inhibit the growth of *Mtb*, although higher amounts may be required for the killing of bacteria. Therefore, there is an urgent need to investigate this potential anti-TB compound. Another study [55], has also pointed out that fisetin might be an ideal compound for investigating anti- Salmonella agents. Much work has been carried out to investigate the immense anti-TB properties of fisetin [56]. The combination of these compounds or their combined use with existing antibiotics should be explored in subsequent studies related to these compounds to increase their efficacy against MDR/TB-causing bacteria. This phytochemical compound could be one of the hitherto unexplored sources of antibiotics, as antibiotic drugs are increasingly becoming unresponsive to bacterial infections [3, 9]. In this respect, the detailed *in vitro Mtb* inhibitory activities of fisetin have been explored using ADME/T, docking, density functional theory, and molecular dynamics simulations. These results are important for understanding the binding affinity and interactions of the compound with the bacterium [57]. The pharmacokinetic profile of fisetin revealed a good ADME/T profile, featuring a suitable molecular weight, strong hydrogen bonding, and Lipinski’s Rule of Five. The improved bioavailability and multitargeted effects of the compounds can be associated with increased polarity and flexible molecular structure, respectively. *Mtb* proteins were investigated to gain more insights into the binding affinity of PanK (type 1), DprE1, EchA6, PknB, PknA, KasA, InhA, and AspAT. Among these, InhA, aspAT, and PknB exhibited high binding affinities. PknB has major implications in various vital bacterial processes such as cell wall biosynthesis, division, and metabolism. In all studies, the data unanimously proved the involvement of PknB in morphological alterations and cell death. Moreover, deletion of the former leads to cell death and plays a very important role in the reactivation of cells during low oxygen tension. Overall, the core importance of PknB in the pathogen has imparted importance to it, making it a promising candidate for the treatment of *Mtb* infection [58].

Aspartate Aminotransferase (AspAT) from Rv3722c is a major contributor to the synthesis of aspartate in *Mtb*. This enzyme connects nitrogen import with survival, as well as the survival of *Mtb* in macrophages [31]. Enoyl acyl carrier protein reductase (InhA) from the NADH-dependent acyl carrier protein reductase family is a major contributor to the synthesis of fatty acids and especially mycolic acids, which are major protective molecules of the *Mtb* cell wall. InhA thus directly functions at the center of *Mtb* survival strategy [59].

The high binding activity of fisetin against PknB, AspAT, and InhA indicates that interference with these key cell survival pathways may have a combined effect on disrupting bacterial cell growth and viability. Fisetin easily bonded with targets in the docking analyses, including to the approved antibiotic isoniazid currently in use. The trends in binding activity were also consistent with studies on natural compounds with structures similar to that of fisetin. For instance, quercetin has been studied as a candidate inhibitor of *Mtb* infection, with a binding energy of -9.61 kcal/mol [60].

The compounds were subjected to molecular dynamics simulations to examine the stability of the complexes. The findings showed that the protein–ligand and co-crystal complexes exhibited distinct dynamic characteristics. Protein Kinase (PknB) was subjected to MD simulation investigation since CCL and fisetin demonstrated remarkable docking efficiency [61, 62].

Using PDB ID 5U94, the binding hotspots of the key were identified by considering residues that consistently interacted with the target. Protein Kinase B (PknB) is essential for the virulence and survival of *Mtb*, according to recent studies on novel TB treatments [63]. The creation of stronger derivatives with greater receptor affinities requires these interactions. The RMSD and RMSF values in the current study showed that the phytocompounds had favorable interactions with the target protein PknB (PDB ID: 5U94). For a simulation duration of up to 100 ns, the system concurrently maintained elasticity and rigidity, according to thorough observations. Whereas DCCM calculations showed better communication between residues, PCA broadened the energy profile of the fisetin system. The significant alterations shown by PCA and cross-correlation studies further demonstrate the dynamic nature of the ligand. Fisetin showed a long-lasting association with Protein Kinase B, was more reactive, and had a high binding energy. Fisetin is another prospective synthon for potential inhibition of important *Mtb* proteins based on the successful completion of docking, DFT calculation, ADME, and MD simulation. In addition, further studies are required to identify new, promising antimicrobial drugs. To provide experimental evidence of the effectiveness of these chemicals for targeted therapy, further laboratory and/or clinical research is required.

## Conclusion

Fisetin’s strong anti-tubercular activity against *Mtb* H37Ra was investigated in this work, which was supported by in vitro and in silico research. The medication showed significant binding affinity toward additional *Mtb* targets in silico, and PknB was found to be the most promising molecular target. Compared to the reference drug (INH), fisetin exhibited superior interaction dynamics and stability, as demonstrated by computational investigations using ADMET, DFT, and molecular dynamics simulations. Fisetin’s capacity to alter important pathways associated with bacterial survival and cell division further highlights its therapeutic potential. These results suggest that fisetin could be a useful natural scaffold for developing novel antitubercular medications. However, further mechanistic and in vivo studies are required to validate its clinical use.

## Supplementary Information

Below is the link to the electronic supplementary material.

Supplementary Material 1

## Data Availability

All data analyzed during this study are available within the manuscript and its supplementary file and can also be found at [https://doi.org/10.6084/m9.figshare.30984100](https:/doi.org/10.6084/m9.figshare.30984100) .
