# Supplementary material for: Integrative computational approaches, molecular docking, and dynamic simulations reveal the antimycobacterial activity of fisetin as a potential inhibitor of Mycobacterium tuberculosis
Source: J Comput Aided Mol Des. 2026 Apr 10;40(1):101. doi: 10.1007/s10822-026-00786-6 (PMC13068679; doi:10.1007/s10822-026-00786-6)
Supplement: Supplementary file 1 — Supplementary Material 1 [file 10822_2026_786_MOESM1_ESM.docx]

**Supplementary Table S1**: Antitubercular activities of Fisetin against Mtb H37Ra

| Compound Name | MIC (µg/mL) | MBC (µg/mL) |
| --- | --- | --- |
| Fisetin | 100 | 200 |
| Isoniazid | 0.156 | 0.156 |
| DMSO | >200 | >200 |

**Supplementary Table S2.** pkCSM prediction of adsorption, distribution, metabolism, excretion and toxicity for Fisetin

| **Property** | **Model Name** | **Predicted Value** | **Unit** |
| --- | --- | --- | --- |
| **Absorption** | Water solubility | **-3.251** | Numeric (log mol/L) |
| **Absorption** | Caco2 permeability | **0.27** | Numeric (log Papp in 10^-6^ cm/s) |
| **Absorption** | Intestinal absorption (human) | **79.391** | Numeric (% Absorbed) |
| **Absorption** | Skin Permeability | **-3.493** | Numeric (log Kp) |
| **Absorption** | P-glycoprotein substrate | **Yes** | Categorical (Yes/No) |
| **Absorption** | P-glycoprotein I inhibitor | **No** | Categorical (Yes/No) |
| **Absorption** | P-glycoprotein II inhibitor | **No** | Categorical (Yes/No) |
| **Distribution** | VDss (human) | **-0.985** | Numeric (log L/kg) |
| **Distribution** | Fraction unbound (human) | **0.242** | Numeric (Fu) |
| **Distribution** | BBB permeability | **-0.886** | Numeric (log BB) |
| **Distribution** | CNS permeability | **-2.249** | Numeric (log PS) |
| **Metabolism** | CYP2D6 substrate | **No** | Categorical (Yes/No) |
| **Metabolism** | CYP3A4 substrate | **No** | Categorical (Yes/No) |
| **Metabolism** | CYP1A2 inhibitior | **Yes** | Categorical (Yes/No) |
| **Metabolism** | CYP2C19 inhibitior | **No** | Categorical (Yes/No) |
| **Metabolism** | CYP2C9 inhibitior | **No** | Categorical (Yes/No) |
| **Metabolism** | CYP2D6 inhibitior | **No** | Categorical (Yes/No) |
| **Metabolism** | CYP3A4 inhibitior | **No** | Categorical (Yes/No) |
| **Excretion** | Total Clearance | **0.477** | Numeric (log ml/min/kg) |
| **Excretion** | Renal OCT2 substrate | **No** | Categorical (Yes/No) |
| **Toxicity** | AMES toxicity | **Yes** | Categorical (Yes/No) |
| **Toxicity** | Max. tolerated dose (human) | **1.069** | Numeric (log mg/kg/day) |
| **Toxicity** | hERG I inhibitor | **No** | Categorical (Yes/No) |
| **Toxicity** | hERG II inhibitor | **No** | Categorical (Yes/No) |
| **Toxicity** | Oral Rat Acute Toxicity (LD50) | **2.312** | Numeric (mol/kg) |
| **Toxicity** | Oral Rat Chronic Toxicity (LOAEL) | **1.981** | Numeric (log mg/kg_bw/day) |
| **Toxicity** | Hepatotoxicity | **No** | Categorical (Yes/No) |
| **Toxicity** | Skin Sensitisation | **No** | Categorical (Yes/No) |
| **Toxicity** | *T.Pyriformis* toxicity | **0.61** | Numeric (log ug/L) |
| **Toxicity** | Minnow toxicity | **2.143** | Numeric (log mM) |

**Supplementary Table S3.** Proteins, co-crystallized ligands, grid center coordinates and box dimensions used for each re-docking complex

| **Protein Name** | **Co-crystallized ligands** | **Grid center coordinates** | **Box dimensions** |
| --- | --- | --- | --- |
| 4BFZ | 2-[4-(4-cyanophenyl)-3-{[4-(pyridin-2-yl)piperazin-1-yl]methyl}phenoxy]-n-methylacetamide **(ZVZ)** | -18.61, -7.1, 10.73 | 20 **Å** |
| 4P8C | Flavin-adenine dinucleotide (**FAD**) | 23.1, -9.1, 0.24 | 20 **Å** |
| 5DUF | (5R,7S)-5-(4-ethylphenyl)-7-(trifluoromethyl)-4,5,6,7-tetrahydropyrazolo[1,5-a]pyrimidine-3-carboxylic acid (**G7A**) | -44.39, 13.76, 8.3 | 20 **Å** |
| 5U94 | 4-{2-(4-amino-1,2,5-oxadiazol-3-yl)-1-ethyl-7-[(3S)-piperidin-3-ylmethoxy]-1H-imidazo[4,5-c]pyridin-4-yl}-2-methylbut-3 -yn-2-ol (**G93**) | -26, -16.12, 1.32 | 20 **Å** |
| 6B2Q | 3-methyl-1-(2-methylpropyl)butyl 4-O-beta-L-gulopyranosyl-beta-D-glucopyranoside (**OBD**) | -27.36, -20.96, -13.94 | 20 **Å** |
| 6R9W | Nicotinamide-adenine-dinucleotide (**NAD**) | 10.13, -37.29, 80.06 | 20 **Å** |
| 6U7A | (2S)-4-(2-aminophenyl)-2-[(E)-({3-hydroxy-2-methyl-5-[(phosphonooxy)methyl]pyridin-4-yl}methylidene)amino]-4-oxobutanoic acid (QOP) | 2.17, 12.7, 4.39 | 20 **Å** |
